# Shedding of Temporary Teeth as to the Process

**Published:** 1889-02

**Authors:** H. H. Schuhman

**Affiliations:** U. of M.


### Shedding of Temporary Teeth as to the Process.

BY H. H. SCHUHMAN, U. OF M.

The most remarkable feature in the process of shedding the temporary teeth is the absorption of their roots.

According to Harris, the process of absorption starts on the interior alveolar plates, then attacks the apices of the roots, first disintegrating that surface nearest the crown of the permanent teeth. If a temporary tooth be removed during the time absorption is taking place, you will find the roots all honeycombed and half dissolved, having somethat the appearance of corrosion.

The cause of absorption is as yet somewhat obscure, and it is surprising to see how greatly the theories of the best authorities of the profession differ on this subject.

If a milk tooth undergoing this process be taken out of the mouth, a loose, spongy substance is found adhering to it, which LaForgue and Bourdet suppose to be an absorbent organ, secreting a fluid capable of dissolving the tooth structure. According to Wedl a fluid is secreted by the cells of this organ which dissolves the hard substance, and referring to the theory held by some he says, "that the cells are of a parasitic nature, that is to say, that the dental substances are eaten up as it were, since the cells absorb the latter," and he remarks that, possibly amoiboid movements may be the occasion of absorption as developed from the peridonteum of the milktooth. According to a very exact and critical examination under the glass, made by Tomes, the surface of this absorbent organ is composed of peculiar compound cells, each of which is made up by a few other cells.

Some say that this process may be due to inflammatory action that excites the cell growth and may as well act as an absorbent, as a reparative. Some thought the cause may be a mechanical force ; others again are of the opinion that this process is caused by atrophy. Prof. Coleman thinks this absorption is due to periosteal secretion.

I shall try now shortly to discuss these six different theories so that we may be more able to judge which seems the correct idea. The six are:

1. Mechanical Force.
2. Atrophy.
3. Coleman's Theory.
4. Wedl's Theory.
5. LaForgue's and Bourdet's.
6. Tomes'.

1. Mechanical Force, that is from pressure of the permanent teeth on the roots of the temporary ones. It is admitted nearly universally by the profession that this theory is incorrect; because sometimes the permanent teeth erupt (they press) and the roots of the milk teeth are not absorbed, so that the permanent tooth erupts either inside or outside of the arch (if the temporary tooth is not removed).

2. Atrophy, is also a wrong theory. If atrophy (starvation of the tissues) were the cause, then not only the roots, but the whole tooth would die.

3. Prof. Coleman thinks that the peroiosteum around the fangs forms the organ of absorption. The character of the periosteal covering becomes changed, its osteoblastic cells, with the power of building up, become osteoclastic, that is, they tear down. This assertion does not seem confirmed when we examine an extracted milktooth undergoing the process of absorption, and find that there is no periosteum, that is, wherever the process of absorption is going on. I don't think it would be likely that the periosteum would first destroy the fangs and then itself, just as little, as I believe in the story told of an alligator eating up a lion, and then the lion the alligator. Much less do I think, could these two processes occur at the same time.

4. Wedl's Theory: Caused by cells of a parasitic nature, with amœboid movement. To me this does not seem to be the real cause. Firstly, such germs are not found after a critical microscopical examination, and secondly, if germs with amœboid power are present, they would attack the surrounding bony structures, as well as the roots of the temporary teeth.

5 and 6. LaForgue's and Bourdet's theory is very much the same as that of Prof. Tomes, and might as well be considered under one head. This theory seems the one "which is thought by the most prominent members of the profession" the more correct one; and in some of our schools it seems to be the only one mentioned.

Prof. Tomes says: "The cementum is first attacked, the dentine disappears next, and the enamel either following or nearly at the same time, wherever the dentine-cementum has been entirely removed, suffers from the same action. When either of these tissues is undergoing this process of absorption, you will find the same characteristic surface as that shown in bone when it is attacked in somewhat the same way and is suffering from a similar action. The surface is covered with deep depressions, having the appearance as if they were brought about by means of some sharp instrument with a semicircular extremity.

The cause of this absorption is a small sack-shaped bundle of cells, each being composed of from two to three to as many as fourteen and fifteen smaller cells. This sack-like body is a secreting organ, brought with its secreting surface toward the apices of the roots of the temporary teeth by the pressure exerted upon it through the permanent tooth.

This secretion was called by Tomes and Harris, "Soluble Ferment" or "Fluid Exudation."

Some may now ask the question, why this fluid does not attack, or does it attack the crowns of the permanent successors?

No, it does not attack them. Like all secreting organs (as the carneous body) it only secretes from *one surface* and that is *toward the temporary tooth.* Another reason is, that the permanent successors are protected by Nasmyth's membrane. This will also answer the question left over from my last paper, why sometimes the temporary tooth is too long retained, sometimes shed too soon and sometimes not shed at all, and if extracted in either of these cases the roots are not at all or only in small degree affected.

If the permanent tooth has a tendency to come too soon, the action of the carneous body will start too soon and if it comes too late will not start until late. In that case, where no permanent successor is coming, the roots are not at all absorbed and such a temporary tooth may remain in the mouth, if kept in a good condition, the life time of the bearer.

There are other reasons why at times the roots of the temporary teeth are not absorbed, though a permanent tooth is following, and those are: that the carneous body may be destroyed by some irritant or destructive agent, as an abscess on the apex of the roots of the temporary tooth. Such a milk tooth might by the permanent one be moved out of place, or *it* may stand and the *permanent tooth* will then have to change its course and come in irregularly.

As the roots of the temporary teeth are absorbed the permanent ones come toward the gum surface, until at length, as it is witnessed every day, the milk teeth drop out of their cavities, minus roots, while the crowns of the permanent ones are seen in *their* places very soon after.

Of course a certain amount of pressure has been equally exerted upon these roots, and as a result, the nutrient vessels passing into the pulp cavity, has been gradually obliterated. The question immediately following this is one left over from my last paper: Do the pulps of the milk teeth die?

Prof. Garretson says: Not at all, or at least not necessarily so. He thinks that the vascularity of the surrounding parts is comparatively great, whilst the requirements of the organ is very small, a sufficient supply is found from the *circulation* in the dentine and cementum.

I cannot quite believe in this theory until I have better reasons and proofs of it. Prof. Garretson tells us that the pulps are not destroyed on account of the *circulation* in the dentine.

As you all know there is no such thing as a *circulation in the*

dentine. It is nourished by infiltration of blood serum (without corpuscles) coming from the periosteum but mostly from the pulp. This supply is just great enough to nourish the dentine, not enough to spare any for the pulp. In fact as you know it just has come from the pulp. Another reason why the pulp is not kept alive by dentine circulation is that plasma alone could not sustain its nutrition. There would have to be a certain amount of corpuscles suspended in it, but which are not present, and if they were, they would have to come from where Prof. Garretson would like to have them go. To me it seems far more likely that the pulps are absorbed.

One of the phenomena of absorption is the pinkish hue we so often see in milk teeth just before being shed. This condition is due to the transparency of the small amount of tooth-substance left showing through the deteriorating bone tissues.

